# Tetra­butyl­ammonium 4-hy­droxy­benzoate dihydrate

**DOI:** 10.1107/S1600536811022823

**Published:** 2011-06-18

**Authors:** Yuan Yang, Yunxia Yang

**Affiliations:** a College of Chemistry and Material Science, Guizhou Normal University, Guiyang 550001, People’s Republic of China; b Key Laboratory of Polymer Materials of Gansu Province, Ministry of Education, College of Chemistry and Chemical Engineering, Northwest Normal University, Lanzhou 730070, Gansu, People’s Republic of China

## Abstract

In the title compound, (*n*-C_4_H_9_)_4_N^+^·C_7_H_5_O_3_^−^·2H_2_O, the carboxyl­ate group is twisted slightly out of the plane of the attached benzene ring, the two C—C—C—O torsion angles being −8.9 (2) and −10.7 (2)°. The anion inter­acts with two water mol­ecules through several O—H⋯O hydrogen bonds, forming wide ribbons along the *a* axis constructed from two anion–water chains. These ribbons are contained between unclosed diamond-like (16.2 × 15.0 Å) channels constructed by four rows of tetra­butyl­ammonium cations, which are arranged along the [011] and [01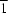] directions.

## Related literature

For related structures of the *p*-hy­droxy­benzoate anion with different cations, see: Marsh & Spek (2001 ▶); Yang *et al.* (2010 ▶). 
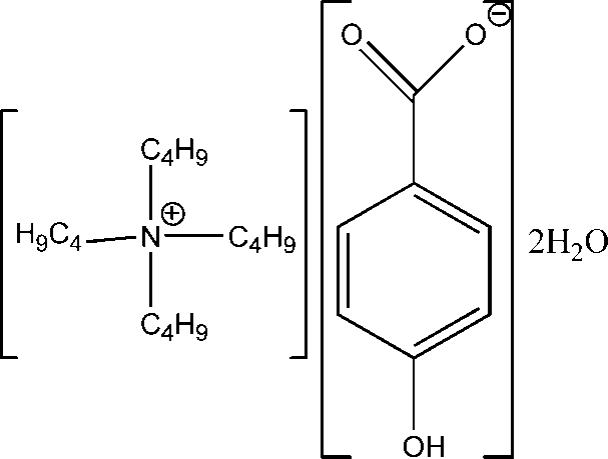


## Experimental

### 

#### Crystal data

C_16_H_36_N^+^·C_7_H_5_O_3_^−^·2H_2_O
*M* *_r_* = 415.60
Monoclinic, 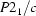

*a* = 10.3679 (1) Å
*b* = 14.9648 (2) Å
*c* = 16.1851 (2) Å
β = 93.128 (1)°
*V* = 2507.43 (5) Å^3^
*Z* = 4
Mo *K*α radiation
μ = 0.08 mm^−1^
*T* = 296 K
0.49 × 0.43 × 0.20 mm

#### Data collection

Bruker SMART APEX diffractometer
Absorption correction: multi-scan (*SADABS*; Sheldrick, 1996 ▶) *T*_min_ = 0.964, *T*_max_ = 0.985
20944 measured reflections
5869 independent reflections
4619 reflections with *I* > 2σ(*I*)
*R*_int_ = 0.018

#### Refinement

*R*[*F* ^2^ > 2σ(*F* ^2^)] = 0.052
*wR*(*F* ^2^) = 0.143
*S* = 1.03
5869 reflections
265 parameters
7 restraints
H atoms treated by a mixture of independent and constrained refinement
Δρ_max_ = 0.54 e Å^−3^
Δρ_min_ = −0.52 e Å^−3^

### 

Data collection: *APEX2* (Bruker, 2007 ▶); cell refinement: *SAINT* (Bruker, 2007 ▶); data reduction: *SAINT*; program(s) used to solve structure: *SHELXS97* (Sheldrick, 2008 ▶); program(s) used to refine structure: *SHELXL97* (Sheldrick, 2008 ▶); molecular graphics: *SHELXTL* (Sheldrick, 2008 ▶); software used to prepare material for publication: *SHELXL97* and *publCIF* (Westrip, 2010 ▶).

## Supplementary Material

Crystal structure: contains datablock(s) I, global. DOI: 10.1107/S1600536811022823/fj2432sup1.cif

Structure factors: contains datablock(s) I. DOI: 10.1107/S1600536811022823/fj2432Isup2.hkl

Supplementary material file. DOI: 10.1107/S1600536811022823/fj2432Isup3.cml

Additional supplementary materials:  crystallographic information; 3D view; checkCIF report

## Figures and Tables

**Table 1:** Hydrogen-bond geometry (Å, °)

*D*—H⋯*A*	*D*—H	H⋯*A*	*D*⋯*A*	*D*—H⋯*A*
O1—H1⋯O2*W*^i^	0.87 (1)	1.75 (1)	2.6125 (17)	174 (2)
O1*W*—H1*WA*⋯O2^ii^	0.86	1.92	2.7660 (18)	168
O1*W*—H1*WB*⋯O2*W*^ii^	0.85	2.22	2.987 (2)	150
O2*W*—H2*WB*⋯O3	0.85	1.80	2.6431 (16)	171
O2*W*—H2*WA*⋯O2^ii^	0.85	1.88	2.7195 (19)	173

Symmetry codes: (i) 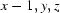; (ii) 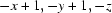.

**Fig. 1.**
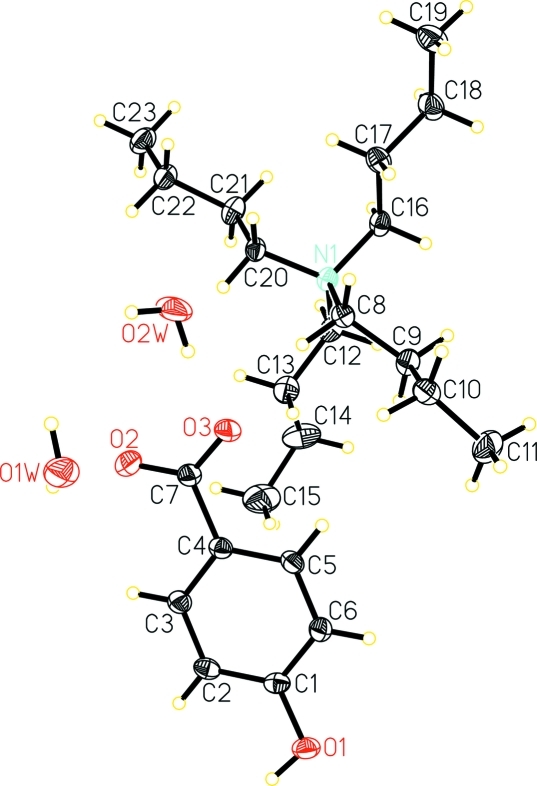
Thermal ellipsoid plot of the title compound at the 30% probability level; hydrogen atoms are drawn as spheres of arbitrary radius.

**Fig. 2.**
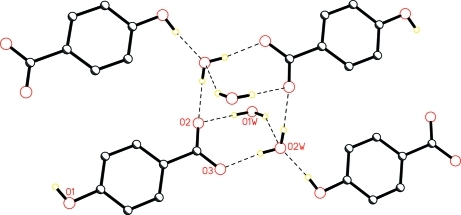
Hydrogen-bonded linking pattern of the wide hydrogen-bonded ribbon in the crystal structure of the title compound.

**Fig. 3.**
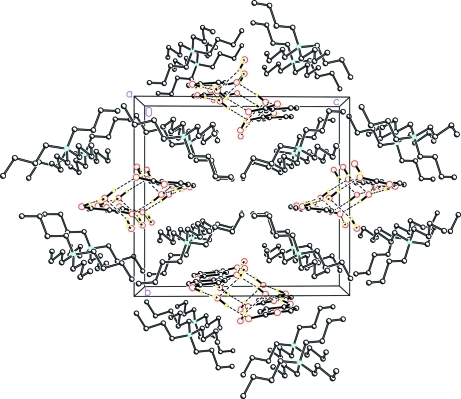
Packing diagram of the title compound; all hydrogen atoms bonded to carbon are omitted for clarity and the cations are represented with the open bonds.

## supplementary crystallographic information

### Comment

*p*-Hydroxybenzoic acid, which can be regarded as a planar aromatic molecule that can form various hydrogen bonds through its different functional groups, has been found to interact with varied cations, such as decyl(trimethyl)ammonium and hexamethonium, to form different crystal structures (Marsh *et al.*, 2001; Yang *et al.*, 2010). Herein we report the crystal structure of tetrabutylammonium *p*-hydroxybenzoate dihydrate, (*n*-C_4_H_9_)_4_N^+^.C_7_H_5_O_3_^-^.2H_2_O, in which the carboxyl group of *p*-hydroxybenzoate anion retorts a small angle of 10.01 (8)° with respect to the phenyl ring and two C—O bonds of the carboxyl group tend to be average (1.264 (2) Å and 1.2553 (18) Å) for the elimination of the proton. The anion makes full use of two independent water molecules to form various O—H···O hydrogen bonds to generate the wide hydrogen-bonded ribbon along the *a* axis (Fig. 2). In addition, four neighboring tetrabutylammonium cations construct un-closed diamond-like channels to contain the hydrogen-bonded ribbons to generate the final stable structure (Fig. 3). Obviously, water molecules, as a kind of linking unit, play an important role in constructing this structure.

### Experimental

*p*-Hydroxybenzoic acid (0.25 mmol, 0.035 g) was dissolved in a water-ethanol (50:100 *v*/*v*) mixture and a 25% aqueous solution of tetrabutylammonium hydroxide was added according to the molar ratio of 1:3 of acid to base. Colorless block crystals separated after several weeks.

### Refinement

All non-hydrogen atoms were refined with anisotropic displacement parameters, and all the hydrogen atoms bonded to carbon were introduced into idealized dispositions. And the hydrogen atoms bonded to oxygen atoms were placed in difference map with fixed distance of 0.86 Å.

### Crystal data

**Table:**

C_16_H_36_N^+^·C_7_H_5_O_3_^−^·2H_2_O	*F*(000) = 920
*M**_r_* = 415.60	*D*_x_ = 1.101 Mg m^−^^3^
Monoclinic, *P*2_1_/*c*	Mo *K*α radiation, λ = 0.71073 Å
Hall symbol: -P 2ybc	Cell parameters from 7508 reflections
*a* = 10.3679 (1) Å	θ = 2.4–27.6°
*b* = 14.9648 (2) Å	µ = 0.08 mm^−^^1^
*c* = 16.1851 (2) Å	*T* = 296 K
β = 93.128 (1)°	Block, colorless
*V* = 2507.43 (5) Å^3^	0.49 × 0.43 × 0.20 mm
*Z* = 4	

### Data collection

**Table:**

Bruker SMART APEX diffractometer	5869 independent reflections
Radiation source: fine-focus sealed tube	4619 reflections with *I* > 2σ(*I*)
graphite	*R*_int_ = 0.018
φ and ω scans	θ_max_ = 27.7°, θ_min_ = 2.0°
Absorption correction: multi-scan (*SADABS*; Sheldrick, 1996)	*h* = −12→13
*T*_min_ = 0.964, *T*_max_ = 0.985	*k* = −19→19
20944 measured reflections	*l* = −21→21

### Refinement

**Table:**

Refinement on *F*^2^	Primary atom site location: structure-invariant direct methods
Least-squares matrix: full	Secondary atom site location: difference Fourier map
*R*[*F*^2^ > 2σ(*F*^2^)] = 0.052	Hydrogen site location: inferred from neighbouring sites
*wR*(*F*^2^) = 0.143	H atoms treated by a mixture of independent and constrained refinement
*S* = 1.03	*w* = 1/[σ^2^(*F*_o_^2^) + (0.062*P*)^2^ + 0.9941*P*] where *P* = (*F*_o_^2^ + 2*F*_c_^2^)/3
5869 reflections	(Δ/σ)_max_ = 0.001
265 parameters	Δρ_max_ = 0.54 e Å^−^^3^
7 restraints	Δρ_min_ = −0.52 e Å^−^^3^

### Special details

**Table:**

Geometry. All e.s.d.'s (except the e.s.d. in the dihedral angle between two l.s. planes) are estimated using the full covariance matrix. The cell e.s.d.'s are taken into account individually in the estimation of e.s.d.'s in distances, angles and torsion angles; correlations between e.s.d.'s in cell parameters are only used when they are defined by crystal symmetry. An approximate (isotropic) treatment of cell e.s.d.'s is used for estimating e.s.d.'s involving l.s. planes.
Refinement. Refinement of *F*^2^ against ALL reflections. The weighted *R*-factor *wR* and goodness of fit *S* are based on *F*^2^, conventional *R*-factors *R* are based on *F*, with *F* set to zero for negative *F*^2^. The threshold expression of *F*^2^ > σ(*F*^2^) is used only for calculating *R*-factors(gt) *etc*. and is not relevant to the choice of reflections for refinement. *R*-factors based on *F*^2^ are statistically about twice as large as those based on *F*, and *R*- factors based on ALL data will be even larger.

### Fractional atomic coordinates and isotropic or equivalent isotropic displacement parameters (Å^2^)

**Table:**

	*x*	*y*	*z*	*U*_iso_*/*U*_eq_	
C1	−0.05049 (13)	0.44078 (9)	0.19491 (9)	0.0322 (3)	
O1	−0.16859 (10)	0.44921 (8)	0.22660 (7)	0.0423 (3)	
H1	−0.2234 (17)	0.4650 (14)	0.1867 (10)	0.063*	
O1W	0.41847 (13)	0.65365 (10)	0.00965 (8)	0.0612 (4)	
H1WA	0.4976	0.6424	−0.0004	0.092*	
H1WB	0.3751	0.6104	−0.0121	0.092*	
N1	0.64105 (11)	0.22051 (7)	0.24724 (7)	0.0305 (3)	
C2	−0.03481 (13)	0.43166 (10)	0.11073 (9)	0.0352 (3)	
H2A	−0.1067	0.4295	0.0738	0.042*	
O2	0.34027 (11)	0.40824 (11)	0.02796 (8)	0.0614 (4)	
O2W	0.65516 (11)	0.49064 (11)	0.11146 (10)	0.0759 (5)	
H2WB	0.5771	0.4792	0.1219	0.114*	
H2WA	0.6579	0.5261	0.0708	0.114*	
C3	0.08817 (14)	0.42582 (10)	0.08181 (9)	0.0351 (3)	
H3A	0.0981	0.4199	0.0253	0.042*	
O3	0.42311 (10)	0.44241 (8)	0.15484 (7)	0.0457 (3)	
C4	0.19687 (13)	0.42867 (9)	0.13567 (9)	0.0309 (3)	
C5	0.17951 (13)	0.43526 (10)	0.22016 (9)	0.0335 (3)	
H5A	0.2513	0.4359	0.2572	0.040*	
C6	0.05727 (14)	0.44095 (10)	0.24987 (9)	0.0357 (3)	
H6A	0.0472	0.4449	0.3065	0.043*	
C7	0.33068 (14)	0.42652 (11)	0.10361 (9)	0.0381 (3)	
C8	0.59979 (14)	0.31219 (9)	0.27669 (8)	0.0310 (3)	
H8A	0.6767	0.3471	0.2907	0.037*	
H8B	0.5527	0.3421	0.2312	0.037*	
C9	0.51640 (16)	0.31144 (10)	0.35062 (9)	0.0383 (3)	
H9A	0.5581	0.2761	0.3947	0.046*	
H9B	0.4337	0.2841	0.3352	0.046*	
C10	0.49506 (16)	0.40589 (11)	0.38123 (10)	0.0412 (4)	
H10A	0.5767	0.4306	0.4026	0.049*	
H10B	0.4633	0.4429	0.3353	0.049*	
C11	0.3990 (2)	0.40765 (15)	0.44849 (12)	0.0632 (5)	
H11A	0.3879	0.4680	0.4668	0.095*	
H11B	0.4308	0.3716	0.4942	0.095*	
H11C	0.3176	0.3845	0.4271	0.095*	
C12	0.52590 (15)	0.15764 (10)	0.24022 (10)	0.0379 (3)	
H12A	0.5545	0.1005	0.2197	0.045*	
H12B	0.4956	0.1478	0.2952	0.045*	
C13	0.41328 (16)	0.18998 (12)	0.18441 (10)	0.0457 (4)	
H13A	0.3893	0.2499	0.2005	0.055*	
H13B	0.4386	0.1921	0.1276	0.055*	
C14	0.29857 (18)	0.12804 (13)	0.19044 (16)	0.0640 (6)	
H14A	0.3222	0.0690	0.1717	0.077*	
H14B	0.2777	0.1232	0.2480	0.077*	
C15	0.1799 (2)	0.15924 (18)	0.13996 (17)	0.0785 (7)	
H15A	0.1104	0.1180	0.1467	0.118*	
H15B	0.1988	0.1621	0.0826	0.118*	
H15C	0.1552	0.2174	0.1585	0.118*	
C16	0.74005 (15)	0.17943 (9)	0.30893 (9)	0.0350 (3)	
H16A	0.7040	0.1788	0.3630	0.042*	
H16B	0.7542	0.1178	0.2931	0.042*	
C17	0.86989 (15)	0.22653 (11)	0.31640 (10)	0.0418 (4)	
H17A	0.9062	0.2290	0.2625	0.050*	
H17B	0.8579	0.2874	0.3352	0.050*	
C18	0.96302 (17)	0.17843 (12)	0.37662 (11)	0.0466 (4)	
H18A	0.9263	0.1761	0.4304	0.056*	
H18B	0.9740	0.1175	0.3578	0.056*	
C19	1.09404 (19)	0.22334 (15)	0.38535 (13)	0.0622 (5)	
H19A	1.1492	0.1905	0.4240	0.093*	
H19B	1.0841	0.2833	0.4050	0.093*	
H19C	1.1318	0.2246	0.3325	0.093*	
C20	0.69736 (14)	0.23417 (10)	0.16349 (8)	0.0327 (3)	
H20A	0.6308	0.2593	0.1259	0.039*	
H20B	0.7665	0.2777	0.1697	0.039*	
C21	0.74994 (17)	0.15052 (11)	0.12424 (10)	0.0427 (4)	
H21A	0.8106	0.1213	0.1632	0.051*	
H21B	0.6796	0.1093	0.1109	0.051*	
C22	0.81703 (17)	0.17376 (12)	0.04617 (10)	0.0464 (4)	
H22A	0.8877	0.2145	0.0600	0.056*	
H22B	0.7564	0.2043	0.0081	0.056*	
C23	0.86954 (18)	0.09191 (13)	0.00355 (11)	0.0525 (4)	
H23A	0.9106	0.1100	−0.0455	0.079*	
H23B	0.7998	0.0518	−0.0112	0.079*	
H23C	0.9314	0.0623	0.0404	0.079*	

### Atomic displacement parameters (Å^2^)

**Table:**

	*U*^11^	*U*^22^	*U*^33^	*U*^12^	*U*^13^	*U*^23^
C1	0.0260 (6)	0.0262 (6)	0.0448 (8)	−0.0008 (5)	0.0035 (6)	0.0035 (6)
O1	0.0271 (5)	0.0484 (6)	0.0519 (7)	0.0028 (5)	0.0067 (5)	0.0059 (5)
O1W	0.0562 (8)	0.0728 (9)	0.0544 (8)	0.0186 (7)	0.0025 (6)	−0.0214 (7)
N1	0.0372 (6)	0.0251 (5)	0.0295 (6)	−0.0007 (5)	0.0055 (5)	0.0017 (4)
C2	0.0258 (7)	0.0369 (7)	0.0420 (8)	−0.0021 (6)	−0.0061 (6)	−0.0032 (6)
O2	0.0365 (6)	0.1066 (11)	0.0417 (7)	0.0004 (7)	0.0071 (5)	0.0015 (7)
O2W	0.0272 (6)	0.1053 (12)	0.0944 (11)	−0.0056 (7)	−0.0034 (6)	0.0513 (10)
C3	0.0313 (7)	0.0401 (8)	0.0335 (7)	0.0001 (6)	−0.0022 (6)	−0.0035 (6)
O3	0.0247 (5)	0.0664 (8)	0.0455 (6)	−0.0036 (5)	−0.0009 (4)	0.0116 (6)
C4	0.0265 (6)	0.0298 (7)	0.0363 (7)	−0.0008 (5)	−0.0001 (5)	0.0042 (5)
C5	0.0283 (7)	0.0371 (7)	0.0346 (7)	−0.0025 (6)	−0.0042 (5)	0.0075 (6)
C6	0.0346 (7)	0.0393 (8)	0.0334 (7)	−0.0011 (6)	0.0030 (6)	0.0056 (6)
C7	0.0281 (7)	0.0473 (9)	0.0391 (8)	−0.0002 (6)	0.0026 (6)	0.0107 (7)
C8	0.0357 (7)	0.0235 (6)	0.0338 (7)	0.0011 (5)	0.0005 (6)	0.0020 (5)
C9	0.0479 (9)	0.0317 (7)	0.0358 (7)	0.0064 (6)	0.0075 (6)	0.0020 (6)
C10	0.0426 (8)	0.0376 (8)	0.0427 (8)	0.0069 (6)	−0.0049 (7)	−0.0084 (6)
C11	0.0745 (13)	0.0632 (12)	0.0530 (11)	0.0182 (10)	0.0151 (10)	−0.0129 (9)
C12	0.0439 (8)	0.0278 (7)	0.0432 (8)	−0.0078 (6)	0.0129 (7)	−0.0030 (6)
C13	0.0452 (9)	0.0481 (9)	0.0438 (9)	−0.0147 (7)	0.0028 (7)	−0.0034 (7)
C14	0.0422 (10)	0.0452 (10)	0.1057 (17)	−0.0099 (8)	0.0135 (10)	−0.0108 (10)
C15	0.0451 (11)	0.0915 (17)	0.0984 (18)	−0.0205 (11)	−0.0005 (11)	−0.0156 (14)
C16	0.0474 (8)	0.0274 (7)	0.0306 (7)	0.0083 (6)	0.0058 (6)	0.0055 (5)
C17	0.0452 (9)	0.0380 (8)	0.0417 (8)	0.0061 (7)	−0.0028 (7)	0.0078 (6)
C18	0.0521 (10)	0.0433 (9)	0.0439 (9)	0.0139 (7)	−0.0024 (7)	0.0067 (7)
C19	0.0495 (10)	0.0715 (13)	0.0642 (12)	0.0145 (9)	−0.0086 (9)	0.0150 (10)
C20	0.0362 (7)	0.0347 (7)	0.0274 (6)	−0.0032 (6)	0.0038 (5)	0.0036 (5)
C21	0.0531 (9)	0.0382 (8)	0.0381 (8)	−0.0055 (7)	0.0131 (7)	−0.0046 (6)
C22	0.0455 (9)	0.0558 (10)	0.0389 (8)	0.0082 (7)	0.0121 (7)	0.0055 (7)
C23	0.0493 (10)	0.0648 (11)	0.0448 (9)	0.0015 (8)	0.0155 (8)	−0.0085 (8)

### Geometric parameters (Å, °)

**Table:**

C1—O1	1.3589 (16)	C12—H12A	0.9700
C1—C2	1.388 (2)	C12—H12B	0.9700
C1—C6	1.390 (2)	C13—C14	1.515 (2)
O1—H1	0.869 (9)	C13—H13A	0.9700
O1W—H1WA	0.8613	C13—H13B	0.9700
O1W—H1WB	0.8528	C14—C15	1.513 (3)
N1—C20	1.5186 (17)	C14—H14A	0.9700
N1—C12	1.5194 (18)	C14—H14B	0.9700
N1—C8	1.5215 (17)	C15—H15A	0.9600
N1—C16	1.5221 (18)	C15—H15B	0.9600
C2—C3	1.385 (2)	C15—H15C	0.9600
C2—H2A	0.9300	C16—C17	1.518 (2)
O2—C7	1.264 (2)	C16—H16A	0.9700
O2W—H2WB	0.8527	C16—H16B	0.9700
O2W—H2WA	0.8463	C17—C18	1.516 (2)
C3—C4	1.3874 (19)	C17—H17A	0.9700
C3—H3A	0.9300	C17—H17B	0.9700
O3—C7	1.2553 (18)	C18—C19	1.515 (3)
C4—C5	1.392 (2)	C18—H18A	0.9700
C4—C7	1.5078 (19)	C18—H18B	0.9700
C5—C6	1.383 (2)	C19—H19A	0.9600
C5—H5A	0.9300	C19—H19B	0.9600
C6—H6A	0.9300	C19—H19C	0.9600
C8—C9	1.5141 (19)	C20—C21	1.518 (2)
C8—H8A	0.9700	C20—H20A	0.9700
C8—H8B	0.9700	C20—H20B	0.9700
C9—C10	1.518 (2)	C21—C22	1.516 (2)
C9—H9A	0.9700	C21—H21A	0.9700
C9—H9B	0.9700	C21—H21B	0.9700
C10—C11	1.515 (2)	C22—C23	1.521 (2)
C10—H10A	0.9700	C22—H22A	0.9700
C10—H10B	0.9700	C22—H22B	0.9700
C11—H11A	0.9600	C23—H23A	0.9600
C11—H11B	0.9600	C23—H23B	0.9600
C11—H11C	0.9600	C23—H23C	0.9600
C12—C13	1.516 (2)		
			
O1—C1—C2	122.41 (13)	C14—C13—H13B	109.6
O1—C1—C6	117.89 (13)	C12—C13—H13B	109.6
C2—C1—C6	119.70 (13)	H13A—C13—H13B	108.1
C1—O1—H1	108.1 (14)	C15—C14—C13	113.23 (18)
H1WA—O1W—H1WB	105.1	C15—C14—H14A	108.9
C20—N1—C12	110.82 (11)	C13—C14—H14A	108.9
C20—N1—C8	106.65 (10)	C15—C14—H14B	108.9
C12—N1—C8	110.40 (11)	C13—C14—H14B	108.9
C20—N1—C16	111.20 (11)	H14A—C14—H14B	107.7
C12—N1—C16	107.33 (11)	C14—C15—H15A	109.5
C8—N1—C16	110.47 (10)	C14—C15—H15B	109.5
C3—C2—C1	119.84 (13)	H15A—C15—H15B	109.5
C3—C2—H2A	120.1	C14—C15—H15C	109.5
C1—C2—H2A	120.1	H15A—C15—H15C	109.5
H2WB—O2W—H2WA	110.6	H15B—C15—H15C	109.5
C2—C3—C4	121.13 (14)	C17—C16—N1	115.34 (11)
C2—C3—H3A	119.4	C17—C16—H16A	108.4
C4—C3—H3A	119.4	N1—C16—H16A	108.4
C3—C4—C5	118.37 (13)	C17—C16—H16B	108.4
C3—C4—C7	120.95 (13)	N1—C16—H16B	108.4
C5—C4—C7	120.67 (12)	H16A—C16—H16B	107.5
C6—C5—C4	121.07 (13)	C18—C17—C16	111.20 (13)
C6—C5—H5A	119.5	C18—C17—H17A	109.4
C4—C5—H5A	119.5	C16—C17—H17A	109.4
C5—C6—C1	119.83 (13)	C18—C17—H17B	109.4
C5—C6—H6A	120.1	C16—C17—H17B	109.4
C1—C6—H6A	120.1	H17A—C17—H17B	108.0
O3—C7—O2	125.68 (14)	C19—C18—C17	112.69 (15)
O3—C7—C4	116.89 (13)	C19—C18—H18A	109.1
O2—C7—C4	117.42 (13)	C17—C18—H18A	109.1
C9—C8—N1	115.04 (11)	C19—C18—H18B	109.1
C9—C8—H8A	108.5	C17—C18—H18B	109.1
N1—C8—H8A	108.5	H18A—C18—H18B	107.8
C9—C8—H8B	108.5	C18—C19—H19A	109.5
N1—C8—H8B	108.5	C18—C19—H19B	109.5
H8A—C8—H8B	107.5	H19A—C19—H19B	109.5
C8—C9—C10	110.53 (12)	C18—C19—H19C	109.5
C8—C9—H9A	109.5	H19A—C19—H19C	109.5
C10—C9—H9A	109.5	H19B—C19—H19C	109.5
C8—C9—H9B	109.5	C21—C20—N1	115.26 (11)
C10—C9—H9B	109.5	C21—C20—H20A	108.5
H9A—C9—H9B	108.1	N1—C20—H20A	108.5
C11—C10—C9	111.34 (15)	C21—C20—H20B	108.5
C11—C10—H10A	109.4	N1—C20—H20B	108.5
C9—C10—H10A	109.4	H20A—C20—H20B	107.5
C11—C10—H10B	109.4	C22—C21—C20	110.57 (13)
C9—C10—H10B	109.4	C22—C21—H21A	109.5
H10A—C10—H10B	108.0	C20—C21—H21A	109.5
C10—C11—H11A	109.5	C22—C21—H21B	109.5
C10—C11—H11B	109.5	C20—C21—H21B	109.5
H11A—C11—H11B	109.5	H21A—C21—H21B	108.1
C10—C11—H11C	109.5	C21—C22—C23	112.66 (15)
H11A—C11—H11C	109.5	C21—C22—H22A	109.1
H11B—C11—H11C	109.5	C23—C22—H22A	109.1
C13—C12—N1	115.04 (12)	C21—C22—H22B	109.1
C13—C12—H12A	108.5	C23—C22—H22B	109.1
N1—C12—H12A	108.5	H22A—C22—H22B	107.8
C13—C12—H12B	108.5	C22—C23—H23A	109.5
N1—C12—H12B	108.5	C22—C23—H23B	109.5
H12A—C12—H12B	107.5	H23A—C23—H23B	109.5
C14—C13—C12	110.37 (15)	C22—C23—H23C	109.5
C14—C13—H13A	109.6	H23A—C23—H23C	109.5
C12—C13—H13A	109.6	H23B—C23—H23C	109.5
			
O1—C1—C2—C3	178.21 (13)	N1—C8—C9—C10	172.91 (12)
C6—C1—C2—C3	−2.2 (2)	C8—C9—C10—C11	173.19 (14)
C1—C2—C3—C4	0.2 (2)	C20—N1—C12—C13	−61.49 (16)
C2—C3—C4—C5	1.6 (2)	C8—N1—C12—C13	56.45 (16)
C2—C3—C4—C7	−177.18 (14)	C16—N1—C12—C13	176.91 (12)
C3—C4—C5—C6	−1.5 (2)	N1—C12—C13—C14	−172.98 (14)
C7—C4—C5—C6	177.33 (14)	C12—C13—C14—C15	176.70 (17)
C4—C5—C6—C1	−0.5 (2)	C20—N1—C16—C17	51.15 (16)
O1—C1—C6—C5	−178.05 (13)	C12—N1—C16—C17	172.52 (12)
C2—C1—C6—C5	2.3 (2)	C8—N1—C16—C17	−67.07 (15)
C3—C4—C7—O3	169.88 (14)	N1—C16—C17—C18	−177.71 (12)
C5—C4—C7—O3	−8.9 (2)	C16—C17—C18—C19	179.72 (15)
C3—C4—C7—O2	−10.7 (2)	C12—N1—C20—C21	−62.15 (16)
C5—C4—C7—O2	170.54 (15)	C8—N1—C20—C21	177.65 (13)
C20—N1—C8—C9	168.00 (12)	C16—N1—C20—C21	57.15 (16)
C12—N1—C8—C9	47.53 (16)	N1—C20—C21—C22	−173.52 (13)
C16—N1—C8—C9	−71.03 (15)	C20—C21—C22—C23	−179.01 (14)

### Hydrogen-bond geometry (Å, °)

**Table:**

*D*—H···*A*	*D*—H	H···*A*	*D*···*A*	*D*—H···*A*
O1—H1···O2W^i^	0.87 (1)	1.75 (1)	2.6125 (17)	174 (2)
O1W—H1WA···O2^ii^	0.86	1.92	2.7660 (18)	168
O1W—H1WB···O2W^ii^	0.85	2.22	2.987 (2)	150
O2W—H2WB···O3	0.85	1.80	2.6431 (16)	171
O2W—H2WA···O2^ii^	0.85	1.88	2.7195 (19)	173

Symmetry codes: (i) *x*−1, *y*, *z*; (ii) −*x*+1, −*y*+1, −*z*.
